# Characterization and Therapeutic Potential of a Novel Bacteriophage Targeting Methicillin‐Resistant *Staphylococcus aureus* Biofilms on Medical Devices

**DOI:** 10.1155/ijm/1281458

**Published:** 2026-08-28

**Authors:** Raphael Kabir Niloy, Shakhinur Islam Mondal, Nurnabi Azad Jewel, Istiaque Zaeem, Mohiminur Rahman Mahin, Daniyal Karim, Mohimenul Haque Rolin, Tahsin Khan, Mohammad Jubair, Mustafizur Rahman, Arzuba Akter

**Affiliations:** ^1^ Department of Genetic Engineering and Biotechnology, Shahjalal University of Science and Technology, Sylhet, Bangladesh, sust.edu; ^2^ Genome Centre, Infectious Diseases Division, International Centre for Diarrhoeal Disease Research Bangladesh, Dhaka, Bangladesh, icddrb.org; ^3^ Department of Biochemistry and Molecular Biology, Shahjalal University of Science and Technology, Sylhet, Bangladesh, sust.edu

**Keywords:** antimicrobial resistance, bacteriophage therapy, bacteriophages, biofilm, genome sequencing, MRSA

## Abstract

Methicillin‐resistant *Staphylococcus aureus* (MRSA) is a major cause of multidrug‐resistant and biofilm‐associated infections, particularly on indwelling medical devices, where conventional antibiotics often exhibit limited efficacy. In this study, we isolated and characterized a novel lytic bacteriophage, SA_SGEB_01, targeting MRSA from hospital wastewater in Bangladesh. Biological characterization demonstrated that SA_SGEB_01 possesses favorable therapeutic properties, including a short latent period (~45 min), a burst size of approximately 91 plaque‐forming units per infected cell, and stability across a broad pH range (3–11) and temperatures up to 45°C. Host range analysis revealed lytic activity against MRSA and methicillin‐resistant *Staphylococcus saprophyticus*. Whole‐genome sequencing showed that SA_SGEB_01 contains a linear double‐stranded DNA genome of 18,031 bp with no detectable lysogeny‐associated, antibiotic resistance, or virulence genes, supporting its therapeutic suitability. Comparative genomic and phylogenetic analyses classified the phage within the genus *Rosenblumvirus* of the family Rountreeviridae. Phage–antibiotic checkerboard assays demonstrated strong synergistic activity between SA_SGEB_01 and ciprofloxacin, with fractional inhibitory concentration index values below 0.5. Importantly, SA_SGEB_01 significantly reduced biofilm biomass in both 24‐ and 48‐h‐old MRSA biofilms and achieved approximately a 2‐log_10_ reduction of viable cells in *in vitro* silicone catheter–associated biofilms, whereas vancomycin did not produce a statistically significant reduction under the same experimental conditions. To our knowledge, this is the first biologically and genomically characterized *S. aureus* phage from Bangladesh with demonstrated activity against MDR MRSA biofilms, highlighting its potential as a promising candidate for controlling device‐associated MRSA infections.

## 1. Introduction

Antimicrobial resistance (AMR) has emerged as a critical global health threat, with projections estimating up to 10 million deaths annually by 2050 if current trends persist [1]. The stagnation of the antibiotic discovery pipeline has further exacerbated this crisis, allowing multidrug‐resistant (MDR) pathogens to proliferate unchecked. Among these, methicillin‐resistant *Staphylococcus aureus* (MRSA) is recognized as a high‐priority pathogen by the World Health Organization due to its prevalence, virulence, and limited treatment options [2–4]. MRSA is responsible for a wide spectrum of hospital‐ and community‐acquired infections, including skin and soft tissue infections, endocarditis, osteomyelitis, pneumonia, and device‐associated infections, with mortality rates reaching as high as 60% in severe cases [5, 6].

The clinical management of MRSA infections is complicated by resistance to *β*‐lactam antibiotics, primarily mediated by the *mecA* gene encoded on the mobile genetic element SCC*mec* (staphylococcal cassette chromosome *mec*). This gene encodes a low‐affinity penicillin‐binding protein that renders most *β*‐lactams ineffective [7]. Although glycopeptides such as vancomycin and oxazolidinones like linezolid remain first‐line therapies, the increasing emergence of vancomycin‐intermediate and vancomycin‐resistant *S. aureus* strains underscores the fragility of current treatment options [8–10].

In addition to intrinsic antibiotic resistance, *S. aureus* exhibits a strong capacity to form biofilms, structured multicellular communities embedded within an extracellular polymeric substance (EPS) matrix [11, 12]. Biofilm formation is a key virulence factor, contributing to more than 80% of chronic and recurrent infections and playing a central role in device‐associated infections such as catheter‐associated urinary tract infections (CAUTIs) [13–15]. Each year, over 30 million urinary catheters are deployed worldwide, with 10%–30% of catheterized patients developing infection [16, 17]. Once established on catheter surfaces, *S. aureus* biofilms contribute to local complications such as encrustation and cystitis while also increasing the risk of bacteremia and septic shock [18].

A major limitation of current antimicrobial strategies is their primary focus on planktonic bacteria, which does not adequately reflect the behavior of biofilm‐associated populations [19]. The EPS matrix restricts antibiotic penetration, alters bacterial metabolic activity, and impairs host immune responses, resulting in persistent infections and frequent treatment failure [20, 21]. Therefore, effective therapeutic approaches must address both planktonic MRSA cells and biofilm‐embedded communities to achieve complete eradication. In this context, bacteriophages offer a promising dual‐action strategy, capable of targeting and lysing MRSA cells while simultaneously penetrating and disrupting biofilm structures.

Bacteriophages, viruses that specifically infect and lyse bacteria, have re‐emerged as promising alternatives to conventional antibiotics [22, 23]. Phages exhibit high host specificity, self‐amplification at infection sites, and minimal disruption of commensal microbiota [24]. Importantly, phages and phage‐derived enzymes can penetrate and dismantle biofilm structures [25–27]. Accumulating evidence also demonstrates that combining phages with antibiotics can produce synergistic effects, enhancing bacterial killing even against antibiotic‐resistant strains [28, 29]. However, such interactions are phage‐ and antibiotic‐specific and require systematic evaluation [28, 29].

Despite growing global interest in phage therapy, there is a paucity of data on well‐characterized *S. aureus* phages from Bangladesh. To date, only limited reports have described the isolation of *S. aureus* phages from this region, without comprehensive biological, genomic, or therapeutic evaluation [30, 31]. This highlights a critical knowledge gap in identifying locally adapted phages capable of effectively targeting MDR MRSA, particularly in biofilm‐associated device infections.

In this study, we report the isolation and comprehensive characterization of a novel lytic *S. aureus* bacteriophage, SA_SGEB_01, from hospital wastewater in Bangladesh. We evaluate its biological properties, genomic features, host range, and stability, along with its antibiofilm efficacy on silicone urinary catheters and its synergistic interaction with ciprofloxacin. Collectively, our findings underscore the therapeutic potential of SA_SGEB_01 as a promising candidate for controlling MDR MRSA and its associated biofilms on medical devices.

## 2. Materials and Methods

### 2.1. Bacterial Strain and Culture Conditions

The MRSA strain MAD918 was used as the host for bacteriophage isolation and propagation. This strain was isolated from Mount Adora Hospital, Sylhet, Bangladesh. Initial identification was performed using the VITEK‐2 Compact system, followed by confirmation in the clinical laboratory through colony morphology, Gram staining, and standard biochemical tests. Molecular confirmation was subsequently achieved by 16S rRNA gene sequencing. Antibiotic susceptibility was determined using the Kirby–Bauer disk diffusion method, with results interpreted according to the 2024 Clinical and Laboratory Standards Institute (CLSI) guidelines (Table 1). Additional strains utilized for host range determination were sourced from internal laboratory collections and are listed in Table S1. All isolates were routinely maintained in Luria–Bertani (LB) broth or agar at 37°C under aerobic conditions. In accordance with ethical standards, this study utilized only anonymized hospital leftovers collected for routine diagnostics; thus, formal ethical approval was not required as no human subjects were involved.

**Table 1:** Antibiogram results indicate that the host strain is multidrug‐resistant. Susceptibility and resistance patterns were interpreted in accordance with CLSI guidelines.

Mechanism of action	Class	Antibiotic	S/R
Inhibits cell wall synthesis (binds PBPs)	Penicillin (*β*‐lactam)	Penicillin G	R
	Penicillinase‐resistant penicillin (*β*‐lactam)	Methicillin	R
		Oxacillin	R
	Cephalosporin (1st generation)	Cefazolin	R
	Cephalosporin/cephamycin (2nd generation)	Cefoxitin	R
Inhibits cell wall synthesis (binds D‐Ala‐D‐Ala)	Glycopeptide	Vancomycin	S
		Teicoplanin	S
Disrupts bacterial cell membrane	Lipopeptide	Daptomycin	S
Inhibits protein synthesis (binds 30S ribosomal subunit)	Tetracycline	Tetracycline	S
		Doxycycline	S
	Glycylcycline	Tigecycline	S
	Aminoglycoside	Gentamicin	S
Inhibits protein synthesis (binds 50S ribosomal subunit)	Macrolide	Erythromycin	R
		Azithromycin	R
	Lincosamide	Clindamycin	R
	Oxazolidinone	Linezolid	S
	Amphenicol	Chloramphenicol	S
Inhibits DNA synthesis (inhibits DNA gyrase/Topoisomerase IV)	Fluoroquinolone	Ciprofloxacin	R
		Levofloxacin	R
		Moxifloxacin	R
Inhibits folate synthesis (inhibits dihydropteroate synthase)	Sulfonamide	Sulfamethoxazole	S
Sequential inhibition of folate synthesis	Combination (sulfonamide + DHFR inhibitor)	Trimethoprim/sulfamethoxazole	S
Inhibits RNA synthesis (inhibits DNA‐dependent RNA polymerase)	Ansamycin	Rifampin	S
Causes DNA damage (via reactive intermediates)	Nitrofuran	Nitrofurantoin	R

### 2.2. Phage Isolation and Purification

Bacteriophage SA_SGEB_01 was isolated from sewage samples collected from the drainage system of Sylhet M.A.G. Osmani Medical College in Sylhet, Bangladesh. Phage enrichment was performed using the MRSA strain MAD918 as the host. To remove large debris, raw samples were centrifuged at 10,000 × *g* for 10 min at 4°C, followed by sterilization of the supernatant through a 0.22‐*μ*m membrane filter. Phage enrichment was conducted by mixing 10 mL of the filtered sewage with 10 mL of double‐strength LB broth and 1 mL of an actively growing culture of the host strain, MRSA MAD918. The mixture was incubated overnight at 37°C with constant shaking. After incubation, the enriched culture was centrifuged at 5000 × *g* for 10 min and filtered again to obtain the crude phage lysate, which was stored at 4°C. The presence of lytic phages was confirmed via spot assays, in which 10 *μ*L of serially diluted lysate was applied to bacterial lawns of strain MAD918. After overnight incubation, the formation of clear lysis zones confirmed lytic activity. A single representative isolate was subsequently selected and designated as SA_SGEB_01.

### 2.3. Plaque Assay and Phage Enumeration

Phage enumeration and plaque morphology assessment were performed using the double‐layer agar (DLA) plaque assay method as described previously [27]. Briefly, mid‐exponential‐phase bacterial host culture was combined with serially diluted phage suspension and mixed with 4 mL of molten LB soft agar containing 0.4% agar, maintained at 50°C. The mixture was then quickly poured onto presolidified LB agar plates containing 1.5% agar and allowed to solidify at room temperature. The plates were incubated overnight at 37°C. After incubation, visible plaques were counted, and plaque characteristics, including plaque size and transparency, were documented. Phage concentration was calculated and reported as plaque‐forming units per milliliter (PFU/mL).

### 2.4. Determination of Multiplicity of Infection (MOI)

The optimal MOI was determined by infecting mid‐logarithmic‐phase host bacteria with varying phage‐to‐bacteria ratios. An overnight bacterial culture was diluted 1:100 in fresh LB broth and incubated at 37°C until mid‐log phase was reached. Phage suspensions were then added at MOIs of 0.001, 0.01, 0.1, 1, 10, 100, and 1000. An uninfected bacterial culture served as a negative control. All cultures were incubated at 37°C with shaking at 180 rpm for 4 h. Following incubation, cultures were centrifuged at 13,000 × *g* for 1 min to remove bacterial debris. The phage titer in the supernatants was subsequently determined using the DLA assay. The MOI that yielded the highest phage titer was defined as the optimal MOI.

### 2.5. One‐Step Growth Curve Analysis

A one‐step growth experiment was performed to determine the latent period and burst size of phage SA_SGEB_01. An early‐log‐phase culture of *S. aureus* MAD918 was infected with the phage at a MOI of 0.1 and allowed to adsorb for 10 min at 37°C. Following adsorption, unbound phages were removed by centrifugation at 13,000 × *g* for 1 min. The pellet was washed three times with fresh LB broth to ensure complete removal of unadsorbed phages. The infected cells were then resuspended in 10 mL of LB broth and incubated at 37°C with shaking at 120 rpm. At defined time intervals (up to 120 min), samples were collected and immediately centrifuged to obtain supernatants. Phage titer was determined using the DLA assay. The latent period and burst size were calculated based on the resulting one‐step growth curve.

### 2.6. pH and Thermal Stability

The stability of phage SA_SGEB_01 under different physicochemical conditions was evaluated by assessing its viability across a range of pH values and temperatures. For pH stability analysis, phage stocks (10^10^ PFU/mL) were diluted 1:10 in SM buffer adjusted to pH values ranging from 3 to 12 and incubated at 37°C for 1 h. Following incubation, the samples were neutralized, serially diluted, and quantified using the DLA assay. For thermal stability assessment, phage aliquots were incubated at 4°C, 25°C, 37°C, 45°C, 50°C, and 60°C for 1 h. After treatment, residual phage infectivity was determined by plaque enumeration using the DLA assay. Phage stability was expressed as the percentage of viable PFU relative to the untreated control.

### 2.7. Host Range Determination

The host range of phage SA_SGEB_01 was evaluated using the spot assay method against a panel of 30 bacterial strains. Briefly, early‐log‐phase bacterial cultures were overlaid onto LB agar plates, after which phage suspensions were spotted onto the bacterial lawns and allowed to dry. The plates were incubated overnight at 37°C and subsequently examined for lytic activity. Zones of lysis were classified as clear (complete lysis), turbid (partial lysis), or absent (no lysis).

### 2.8. Phage Genome Sequencing and Genome Analysis

#### 2.8.1. Phage DNA Extraction, Sequencing, and Genome Assembly

Phage genomic DNA was extracted from high‐titer lysates using a polyethylene glycol (PEG)–based precipitation method with minor modifications. Briefly, phage lysates were concentrated overnight at 4°C using PEG 8000 (10%) and NaCl (1 M), followed by centrifugation at 12,000 × *g* for 2 h. The pellet was resuspended in 5 mM MgSO_4_ and treated with DNase I and RNase A (20 *μ*g/mL each) at 37°C for 1 h to remove host nucleic acids. Phage capsids were disrupted using Proteinase K, SDS, and EDTA, followed by incubation at 60°C for 1 h. Viral DNA was purified using the PureLink Viral RNA/DNA Mini Kit according to the manufacturer′s instructions. Whole‐genome sequencing was performed at the Child Health Research Foundation (CHRF) using the Illumina NextSeq 2000 platform. Raw reads were quality‐filtered using FastP v1.0.1 [32] and assembled *de novo* using MEGAHIT v1.2.9, followed by refinement with Pilon v1.24 [33, 34]. Genome completeness and quality were assessed using QUAST v5.3.0 and CheckV v1.0.3 [35, 36].

#### 2.8.2. Genome Annotation and Comparative Analysis

Genome annotation was performed using Pharokka v1.8.2 with open reading frame (ORF) prediction by Prodigal [37, 38] and functional annotation based on the PHROGs database [39]. Phage lifestyle was predicted using PhaTYP [40]. Circular genome visualization was generated using pyCirclize (https://moshi4.github.io/pyCirclize/). Taxonomic classification was determined according to current ICTV guidelines using ViPTree, taxMyPhage, and VIRIDIC based on proteomic and intergenomic similarities [41–43].

For comparative genomic analysis, complete *Staphylococcus* phage genomes were retrieved from the INPHARED database (https://github.com/RyanCook94/inphared) (accessed December 2025). Redundant genomes were removed using CD‐HIT with a 95% identity threshold [44] and reannotated using Pharokka [37]. The PHROGs database was utilized for functional annotation [38, 39]. Protein sequences were clustered into protein families (phams) employing PhaMMseqs [45] and PhamClust [46]. Intergenomic similarities were further assessed using VIRIDIC [43].

#### 2.8.3. Phylogenetic Analysis

Whole‐genome phylogenetic analysis was conducted using the VICTOR web server (https://ggdc.dsmz.de/victor.php) [47] based on Genome‐BLAST Distance Phylogeny (GBDP). Phylogenetic trees were constructed using the FastME algorithm and supported by 100 pseudobootstrap replicates. Single‐gene phylogenies were generated utilizing the ETE3 pipeline (v3.1.3) via the GenomeNet server [48]. Amino acid sequences were aligned with MAFFT (v6.861b) and trimmed with trimAl (v1.4.rev6) [49, 50]. Phylogenetic trees were reconstructed using IQ‐TREE (v1.5.5) with model selection by ModelFinder [51]. Branch support was assessed using SH‐like approximate likelihood ratio tests.

#### 2.8.4. Identification of Lysis Module Components

Lysis‐associated proteins, including endolysins and holins, were identified by screening PhaMMseqs clusters using the keywords “endolysin,” “amidase,” and “holin.” Conserved domains were predicted using PfamScan (v1.6) (https://github.com/aziele/pfam_scan) and validated through the InterPro web server [52]. Phylogenetic relationships of the identified proteins were analyzed using the methods described above.

### 2.9. Phage–Antibiotic Synergy Assay

The combined antibacterial efficacy of phage SA_SGEB_01 and ciprofloxacin was evaluated using a checkerboard assay. Phage suspensions (10^2^–10^8^ PFU/mL) and ciprofloxacin (0.125–64 *μ*g/mL) were serially diluted and combined with bacterial inocula adjusted to 10^6^ CFU/mL in a final volume of 160 *μ*L/well. Following incubation, bacterial growth was quantified by measuring optical density at 600 nm (OD_600_). Growth inhibition percentages were calculated and visualized as heatmaps. Synergistic interactions between phage and antibiotic treatments were evaluated by calculating the fractional inhibitory concentration index (FICI).

### 2.10. Biofilm Reduction Assay

Biofilms of *S. aureus* MAD918 were established by diluting overnight bacterial cultures 1:100 in tryptic soy broth (TSB) supplemented with 1% glucose. Here, biofilms were allowed to develop for either 24 or 48 h prior to phage treatment, which were designated as immature‐stage and mature‐stage biofilms, respectively. Aliquots (200 *μ*L) were dispensed into 96‐well polystyrene microtiter plates and incubated statically at 37°C for 24 h (early stage or immature stage) or 48 h (mature stage). Following incubation, planktonic cells were removed, and the wells were washed three times with sterile SM buffer. Phage suspensions prepared in TSB were then added to the wells (200 *μ*L), whereas SM buffer–treated wells served as negative controls. Plates were incubated at 37°C for 4 h. After treatment, the wells were washed and stained with 0.01% crystal violet for 15 min. Excess stain was removed by washing, and the plates were air‐dried. The bound crystal violet was solubilized using 30% acetic acid, and biofilm biomass was quantified by measuring absorbance at 595 nm. Antibiofilm activity was assessed by comparing biofilm biomass in phage‐treated wells with that of untreated control wells following the same treatment period. Again, this activity was quantified by measuring OD_595_; viable bacterial counts (CFU) were not determined in this assay.

### 2.11. Biofilm Reduction on Silicone Catheter Segments

The antibiofilm activity of phage SA_SGEB_01 on clinically relevant surfaces was evaluated using silicone Foley catheter segments. Sterile silicone catheters were aseptically cut into 1.5‐cm segments and transferred into sterile 100‐mL conical flasks containing TSB supplemented with glucose. The medium was inoculated with *S. aureus* MAD918 to a final concentration of approximately 1 × 10^7^ CFU/mL and incubated statically at 37°C for 24 h to allow biofilm formation on the catheter surfaces. Following incubation, catheter segments were washed three times with SM buffer to remove planktonic cells and transferred into microcentrifuge tubes containing SM buffer (control), vancomycin (5 mg/mL), or phage SA_SGEB_01 (1 × 10^9^ PFU/mL). After incubation at 37°C for 4 h, the segments were washed, and adherent biofilms were detached by repeated vortexing and sonication in SM buffer. The recovered bacterial suspensions were serially diluted and plated for CFU enumeration. The efficacy of phage treatment was assessed by comparing viable bacteria from treated catheter‐associated biofilms with those from untreated control biofilms after the same incubation period.

### 2.12. Statistical Analysis

All experiments were performed in triplicate, and data are presented as mean ± standard deviation. Statistical analyses and graphical representations were conducted using the R programming environment. Statistical significance was considered at *p* < 0.05, and the specific tests used are described in the corresponding figure legends.

## 3. Results

### 3.1. Isolation and Purification of Bacteriophage

**Figure 1:**
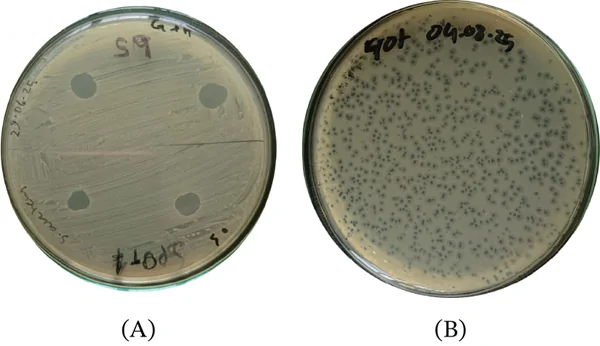
Spot test and phage plaque morphology. (A) Spot test and (B) plaques formed by the phage SA_SGEB_01 on the culture medium of the host bacterium MAD918.

Bacteriophage SA_SGEB_01 was successfully isolated from sewage samples using *S. aureus* MAD918 as the host strain. Initial screening by spot‐on‐lawn assay produced a distinct zone of lysis, indicating the presence of a lytic bacteriophage (Figure 1A). Following purification and propagation, plaque morphology was further examined using the DLA assay. SA_SGEB_01 produced clear, circular plaques with an average diameter of approximately 2 mm (Figure 1B and Figure S1). The clear plaque morphology suggests a strictly lytic lifestyle, supporting the potential suitability of SA_SGEB_01 for therapeutic applications.

### 3.2. MOI

**Figure 2:**
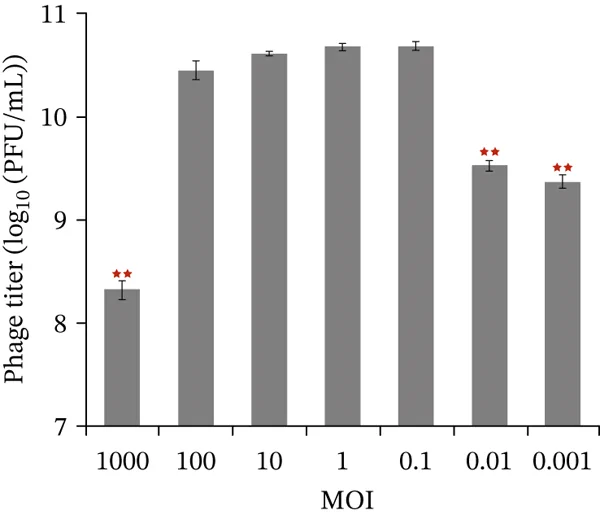
Optimal multiplicity of infection for the phage SA_SGEB_01. After 4 h of cultivation, 100 *μ*L is mixed with 200 *μ*L of host bacteria and allowed to stand for 10 min, and the titer is determined by the DLA method. Statistical significance was determined using one‐way ANOVA followed by Tukey′s multiple comparisons test. Significance annotations are shown relative to the MOI 0.1 group for clarity. Significance levels are indicated as  ^∗^ *p* < 0.05 and  ^∗∗^ *p* < 0.01.

The optimal MOI for phage SA_SGEB_01 was determined by comparing phage yields obtained at different phage‐to‐host ratios. Phage titer generated at MOIs of 100, 10, 1, and 0.1 showed no significant differences (*p* > 0.05). However, a significant reduction in phage production was observed at an MOI of 0.01 (*p* < 0.05) (Figure 2). Based on these findings, an MOI of 0.1 was selected as the optimal condition for subsequent experiments.

### 3.3. Growth Kinetics of Phage SA_SGEB_01

**Figure 3:**
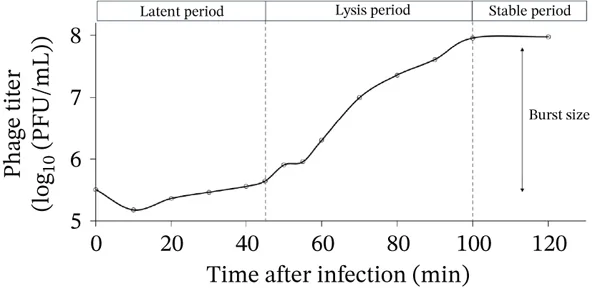
One‐step growth curve of the phage. There is a 45 min latent period followed by almost 50 min of the lysis period. Subsequently, there is a plateau indicating a stable period. The burst size was calculated to be ~91 PFU/CFU.

The replication kinetics of phage SA_SGEB_01 were evaluated using a one‐step growth curve assay, which demonstrated a typical lytic replication pattern consisting of latent, rise, and plateau phases (Figure 3). An initial latent period of approximately 45 min was observed, during which no significant increase in extracellular phage particles was detected due to phage adsorption and intracellular replication within the host cells. This was followed by a rapid rise phase characterized by a sharp increase in phage titer resulting from the release of newly produced virions. The rise phase continued until approximately 100 min after infection, after which the phage titer reached a plateau, indicating completion of the lytic cycle. Based on the one‐step growth analysis, the average burst size of SA_SGEB_01 was estimated to be approximately 91 plaque‐forming units (PFU) per infected bacterial cell.

### 3.4. Temperature and pH Stability

**Figure 4:**
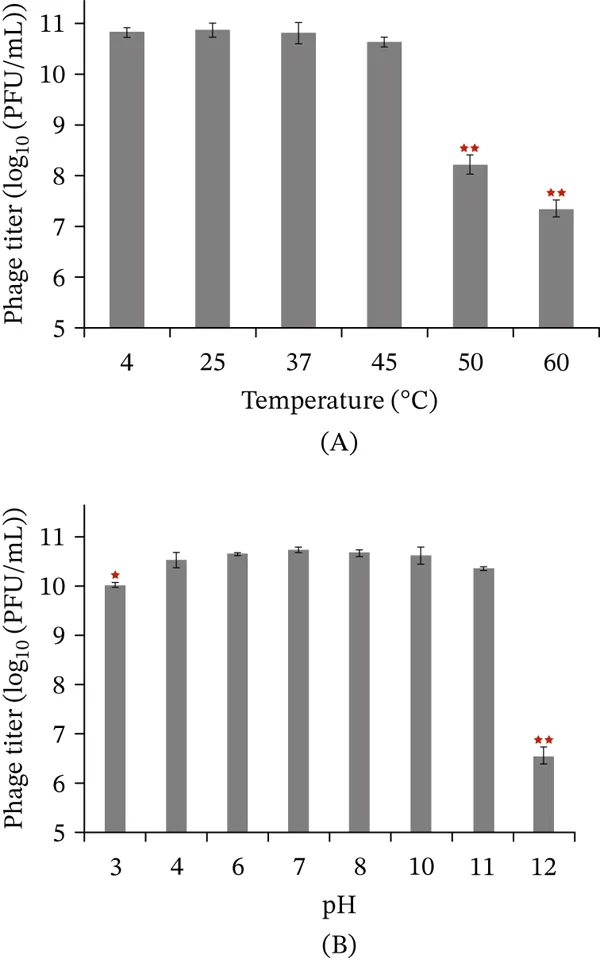
Stability profile of the phage. (A) Temperature stability after 1‐h treatment at 4°C, 25°C, 37°C, 45°C, 50°C, and 60°C. (B) pH stability of phage SA_SGEB_01 after 1‐h treatment under various pH conditions. Statistical significance was determined using one‐way ANOVA followed by Dunnett′s multiple comparisons test, with 4°C (temperature experiment) or pH 7 (pH experiment) as the control. Significance levels are indicated as  ^∗^ *p* < 0.05 and  ^∗∗^ *p* < 0.01.

The stability of phage SA_SGEB_01 under different physicochemical conditions was evaluated by exposing the phage to varying temperatures and pH levels (Figure 4). Temperature stability analysis demonstrated that SA_SGEB_01 remained highly stable between 4°C and 45°C, with no significant reduction in phage titer observed across this range (*p* > 0.05). In contrast, exposure to elevated temperatures of 50°C and 60°C resulted in a significant loss of phage viability (*p* < 0.001) (Figure 4A).

Similarly, pH stability assays revealed that SA_SGEB_01 retained infectivity over a broad pH range from 3 to 11, with no significant differences in phage titer detected (*p* > 0.05). The highest titer was observed at pH 7 and 8, reaching 10.74 and 10.68 log PFU/mL, respectively. However, exposure to highly alkaline conditions (pH 12) caused a significant reduction in phage viability (*p* < 0.001) (Figure 4B). Overall, these findings indicate that SA_SGEB_01 possesses substantial stability across a wide range of environmental conditions.

### 3.5. Host Range Determination

The host range of phage SA_SGEB_01 was evaluated against a panel of 30 bacterial strains using spot assays (Table 2). Clear lytic activity was observed against two strains, including the host strain *S. aureus* MAD918 and a methicillin‐resistant *Staphylococcus saprophyticus* isolate. In addition, several strains exhibited turbid or partial lysis, whereas no lytic activity was detected against the remaining isolates. These findings suggest that SA_SGEB_01 possesses a moderate but clinically relevant host range, with lytic activity extending beyond *S. aureus* to include coagulase‐negative staphylococci.

**Table 2:** Host range of the *S. aureus* phage SA_SGEB_01.

Strain	Species	Plaque morphology
MAD918	*Staphylococcus aureus* (isolation host)	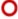
ATCC_25923	*Staphylococcus aureus*	X
MAD557	*Staphylococcus aureus*	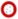
MAD895	*Staphylococcus aureus*	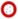
MAD371	*Staphylococcus auricularis*	X
MAD342	*Staphylococcus epidermidis*	X
ICD002	*Staphylococcus haemolyticus*	X
MAD286	*Staphylococcus saprophyticus*	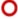
ATCC_12156	*Acinetobacter baumannii*	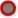
ICD005	*Acinetobacter baumannii*	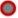
MAD807	*Acinetobacter baumannii*	X
MAD994	*Acinetobacter baumannii*	X
MAD546	*Acinetobacter lwoffii*	X
ICD003	*Enterobacter cloacae* complex	X
EF_BMB_02	*Enterococcus faecalis*	X
ATCC_25922	*Escherichia coli*	X
ICD004	*Escherichia coli*	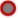
EC_NR_01	*Escherichia coli*	X
MAD685	*Klebsiella aerogenes*	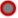
ICD001	*Klebsiella pneumoniae*	X
MAD058	*Klebsiella pneumoniae*	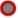
MAD748	*Klebsiella pneumoniae*	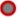
MAD787	*Klebsiella pneumoniae*	X
ATCC_27853	*Pseudomonas aerugenosa*	X
cc2	*Salmonella enterica*	X
MD1	*Salmonella enterica*	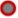
SAU_S15	*Salmonella enterica*	X
ATCC_14028	*Salmonella enterica* serotype Typhi	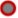
ST_BMB_01	*Salmonella enterica* serotype Typhi	X
VC_BMB_01	*Vibrio cholerae*	X

*Note:* Morphology (DST): Clear 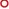, moderate 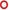, and foggy 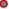.

### 3.6. Phage Genome Sequencing and Genome Analysis

#### 3.6.1. Genome Features and Functional Annotation

**Figure 5:**
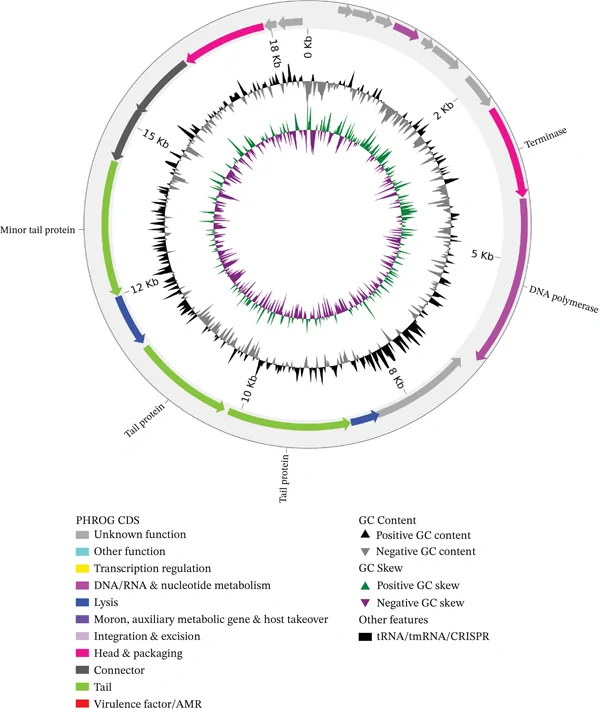
Genome map of SA_SGEB_01 using pyCirclize in the Pharokka pipeline. Labeling of the putative coding sequence regions is in the outer ring. The coding sequences were colored according to their functional modules, and the transcriptional direction is shown by the arrows. GC content has been represented by the second inner ring, and GC skew has been shown by the innermost ring.

Whole‐genome sequencing revealed that SA_SGEB_01 possesses a linear double‐stranded DNA genome of 18,031 bp with a GC content of 29% and a coding density of 94.24% (Table 3). CheckV analysis confirmed that the genome was complete and of high quality. A total of 20 ORFs were predicted, encoding proteins associated with DNA replication, virion structure, and host lysis (Figure 5).

**Table 3:** Genome characteristics of SA_SGEB_01.

Parameter	SA SGEB_01
Genomic size (bp)	18,031
GC content (%)	29
Coding density (%)	94.24
Proteins (*n*)	20
Resistant genes	0
Bacterial virulence genes	0
Lytic proteins	1
Taxonomy (virus superkingdom)	
Clade	Duplodnaviria
Kingdom	Heunggongvirae
Phylum	Uroviricota
Class	Caudoviricetes
Family	Rountreeviridae
Subfamily	Rakietenvirinae
Genus	*Rosenblumvirus*
Species	*Rosenblumvirus SCH1*

Genome annotation identified a canonical two‐component lysis module consisting of a Class IV holin (Phage_holin_4_1) and a modular endolysin containing an N‐terminal CHAP catalytic domain and a C‐terminal SH3‐type cell wall–binding domain (SH3_5). Importantly, no genes associated with lysogeny, antibiotic resistance, or bacterial virulence were detected, supporting the therapeutic suitability of SA_SGEB_01.

#### 3.6.2. Comparative Genomics and Taxonomic Classification

**Figure 6:**
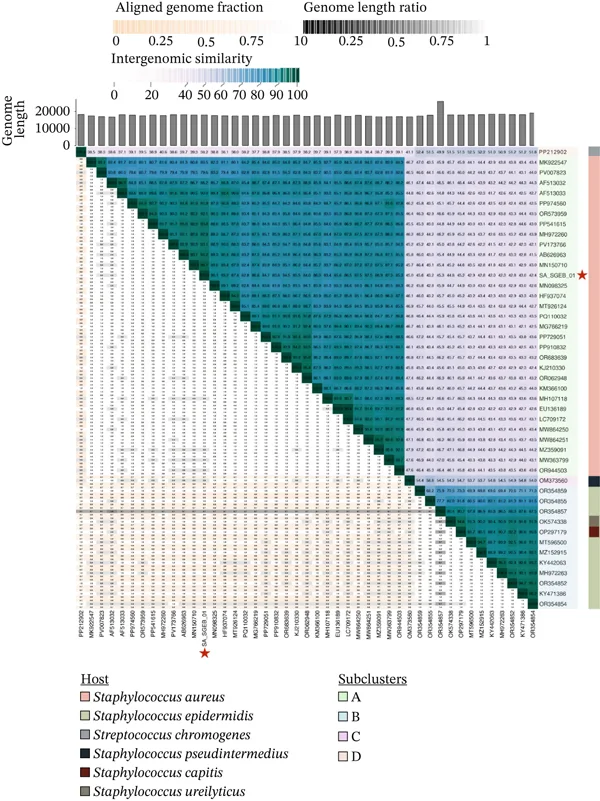
VIRIDIC heatmap of the 45 *Staphylococcus* phages. Intergenomic similarities among the phages are shown on the right side, using a color scale and an ANI score; more distinct clusters are observed than in the dot plot. The aligned genome fraction and genome length ratio values are shown on the left side with their corresponding scales. Overlayed is the color coding of the four subclusters.

Comparative genomic and phylogenetic analyses consistently placed SA_SGEB_01 within the family Rountreeviridae. Intergenomic similarity analysis revealed 96.3% nucleotide identity with *Staphylococcus* phage *SCH1* (indicated as KY000084 in the plot), supporting its classification within the genus *Rosenblumvirus* and assignment to the species *Rosenblumvirus SCH1* (Figure 6 and Figure S2).

#### 3.6.3. Pangenome and Phylogenetic Analysis

**Figure 7:**
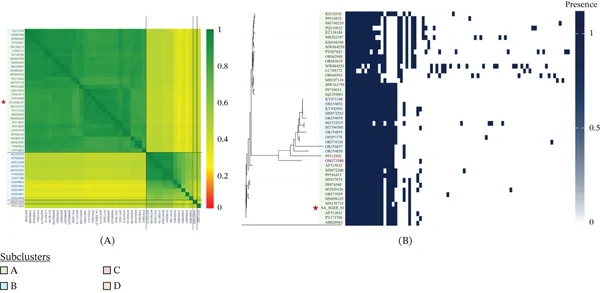
Pangenome characteristics of Cluster 5 *Staphylococcus* phages. (A) PEQ heatmap shows that 45 genomes were clustered in a cluster (Cluster 5) with SA_SGEB_01. This cluster was further divided into four subclusters. Subcluster A contained all the phages from the genus *Rosenblumvirus*, whereas Subclusters B and C consisted of phages of the *Andhravirus* genus. (B) Gene presence/absence matrix from pangenome analysis of 45 *Staphylococcus* phage genomes with PhamClust. Each row presents the gene profile for an isolate.

Pangenome analysis of 625 nonredundant *Staphylococcus* phage genomes grouped SA_SGEB_01 within Cluster 5, which contained 45 closely related lytic phages (Figure S3). Cluster 5 genomes ranged from 16 to 26 kb with an average coding density of 93.59% (Figure 7A). A total of 82 gene families (phamilies) were identified, including four core genes shared across all members (Figure 7B).

**Figure 8:**
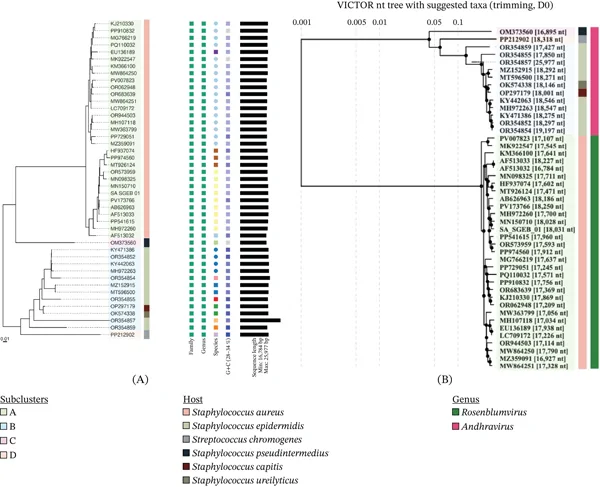
Phylogenetic relationships of Cluster 5 *Staphylococcus* phages. (A) Phylogenetic tree based on the whole genome generated by VICTOR. It predicts that all phages belong to the same family and genus and are classified into 14 species. Additionally, subclusters are annotated in different colors, and the host is annotated with the corresponding color strip, as indicated in the legend. (B) ViPTree proteome–based phylogenetic tree with other phages belonging to Cluster 5. Overlayed is the color coding of the four subclusters. The color strips indicate the host and the actually assigned genus of the phages.

**Figure 9:**
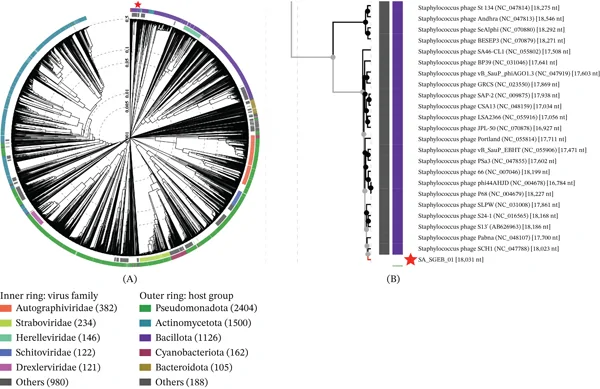
Proteomic tree analysis reveals that SA_SGEB_01 has the highest similarity with the viruses from the genus *Rosenblumvirus.* (A) Circular proteomic tree. (B) Rectangular proteomic tree.

Whole‐genome phylogenetic analysis using the VICTOR platform consistently clustered SA_SGEB_01 within the family Rountreeviridae. Proteomic phylogenetic analysis using ViPTree further identified *Staphylococcus* phage *SCH1* as the closest evolutionary relative of SA_SGEB_01 (Figures 8 and 9).

#### 3.6.4. Diversity of Lysis‐Associated Proteins

**Figure 10:**
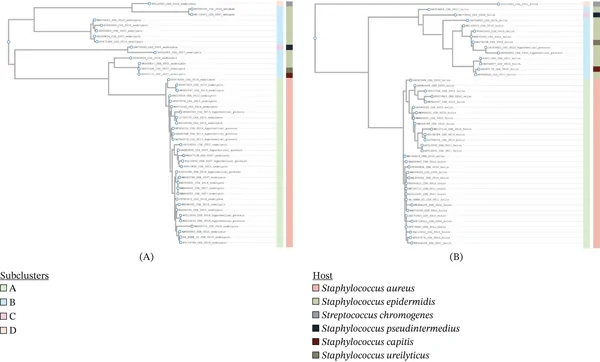
The phylogenetic tree of holin and endolysin sequences derived from *Staphylococcus* phages was constructed using the ETE3 framework hosted on GenomeNet. In the resulting phylogenetic tree, the initial color strip adjacent to the terminal nodes identifies subclusters within Cluster 5 phages, whereas the subsequent strip denotes the specific cluster of the associated hosts. (A) Analysis revealed that holins generally form discrete clades and show less versatility. Furthermore, all holins from the genus *Rosenblumvirus* formed a monophyletic clade under strong evolutionary pressure. (B) Comparative analysis indicates that endolysins exhibit significantly higher evolutionary plasticity than holins, manifesting more heterogeneous and versatile clades. This divergence suggests a broader range of adaptations within the endolysin domain architecture compared with the relatively conserved holins.

Comparative analysis of Cluster 5 phages identified 45 endolysins and 45 holin sequences. Endolysins showed substantial sequence and domain diversity and were grouped into five phamilies. The dominant group contained CHAP‐domain endolysins with SH3‐type cell wall–binding domains, whereas smaller groups encoded amidase‐type enzymes. In contrast, holins were highly conserved, and all belonged to the Phage_holin_4_1 superfamily. Phylogenetic analysis demonstrated greater diversity among endolysins than holins, suggesting stronger evolutionary constraints on holin function (Figure 10A,B).

### 3.7. Synergistic Antibacterial Activity of Phage SA_SGEB_01 and Ciprofloxacin

**Figure 11:**
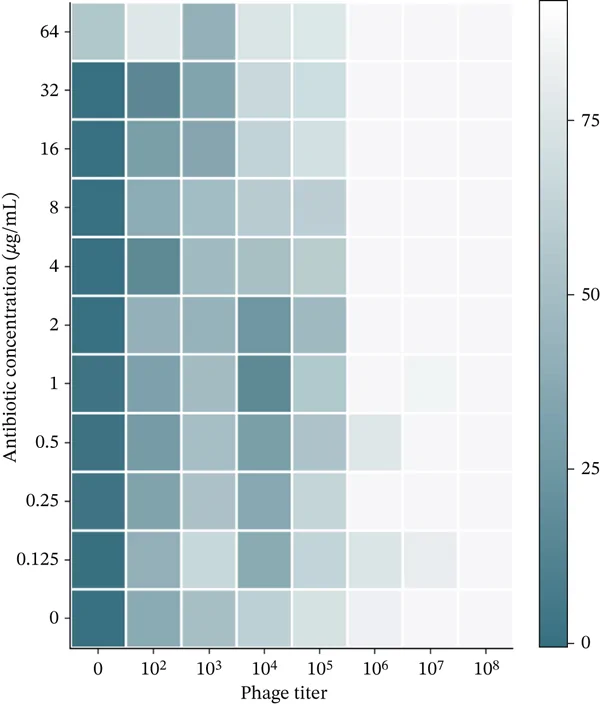
Checkerboard analysis of ciprofloxacin and phage SA_SGEB_01. Antibiotic concentrations at the ordinate, ranging from 0.125 to 64 *μ*g/mL, and phage concentrations at the abscissa. Growth inhibition is shown as a heatmap.

The antibacterial activity of phage SA_SGEB_01 in combination with ciprofloxacin was evaluated using a checkerboard assay (Figure 11). Phage treatment alone produced a dose‐dependent reduction in bacterial growth, with approximately 87% inhibition observed at 10^6^ PFU/mL and up to 95%–96% inhibition at 10^7^ and 10^8^ PFU/mL. In contrast, ciprofloxacin monotherapy showed limited antibacterial activity, with a maximum growth reduction of approximately 39% even at the highest tested concentration, consistent with the ciprofloxacin‐resistant phenotype of *S. aureus* MAD918.

Notably, combined treatment with phage and ciprofloxacin resulted in significantly enhanced antibacterial activity compared with either treatment alone. At a phage concentration of 10^6^ PFU/mL, the combination achieved more than 95% inhibition across a broad range of ciprofloxacin concentrations, including levels at which the antibiotic alone was largely ineffective. FICI values were consistently below 0.5, confirming a synergistic interaction between SA_SGEB_01 and ciprofloxacin. These findings indicate that phage treatment can substantially enhance the antibacterial efficacy of ciprofloxacin against resistant MRSA strains.

### 3.8. Antibiofilm activity of Phage SA_SGEB_01

**Figure 12:**
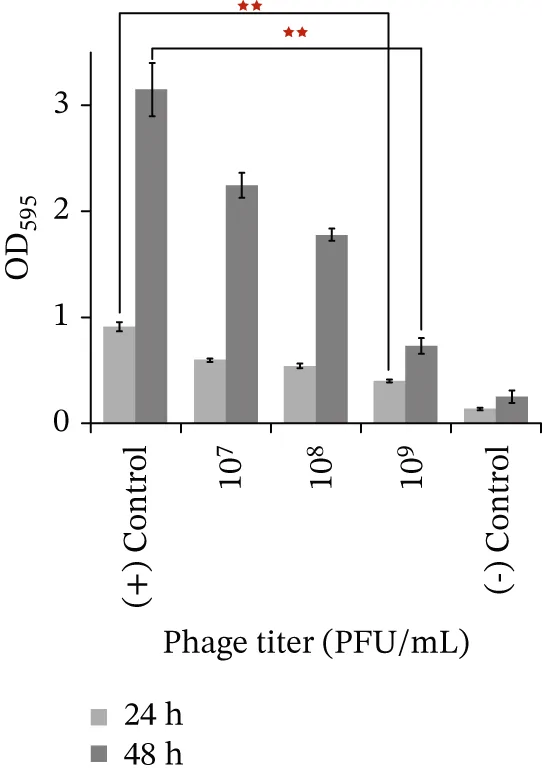
Antibiofilm assay of SA_SGEB_01. The assay shows a significant reduction (*p* < 0.05) in the 24‐ and 48‐h‐old biofilms. Statistical significance was determined using one‐way ANOVA with Tukey′s multiple comparisons test. The significance bar denotes the comparison between the untreated positive control and the 10^9^ PFU/mL treatment. Asterisks indicate adjusted *p* values (*p* < 0.05 and *p* < 0.01).

The antibiofilm activity of phage SA_SGEB_01 was evaluated using a crystal violet–based microtiter plate assay against MRSA strain MAD918. Phage treatment significantly reduced biofilm biomass in both 24‐h‐old (early‐stage) and 48‐h‐old (mature‐stage) MRSA biofilms in a dose‐dependent manner (Figure 12). At the highest phage concentration tested (10^9^ PFU/mL), biofilm biomass was reduced by approximately 76.6% in 48‐h‐old biofilms (*Δ*OD_595_ = 2.41) and 56.3% in 24‐h‐old biofilms (*Δ*OD_595_ = 0.51) relative to the untreated positive controls. Although the reduction was comparatively higher in 48‐h‐old biofilms, significant decreases in biomass were observed in both conditions compared with untreated controls.

### 3.9. Reduction of MRSA Biofilms on Silicone Catheter Surfaces

**Figure 13:**
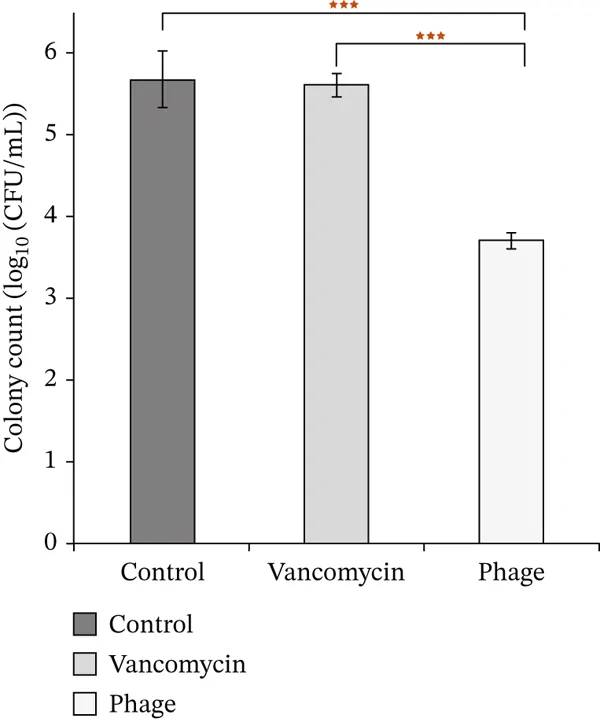
Treatment of MRSA biofilms established on catheter sections. Biofilms were grown on 1.5‐cm polyurethane catheters for 24 h in vitro and then treated with either vancomycin (5 mg/mL) or SA_SGEB_01 (10^9^ PFU/mL). Viable CFUs were determined on agar plates following treatment. Significance levels are indicated as ∗∗∗(*p* < 0.001).

The antibiofilm activity of SA_SGEB_01 was further evaluated against MRSA biofilms formed on silicone Foley catheter segments under clinically relevant conditions (Figure 13). Phage treatment resulted in an approximately 2.0‐log_10_ reduction in viable MRSA cells compared with untreated controls. In contrast, vancomycin treatment did not produce a statistically significant reduction in viable bacterial counts.

Moreover, SA_SGEB_01 treatment produced an approximately 1.9‐log_10_ greater reduction in viable cells compared with vancomycin treatment. Statistical analysis using one‐way ANOVA demonstrated a significant overall effect of treatment on bacterial viability (*p* = 0.000296). These results indicate that SA_SGEB_01 effectively reduced catheter‐associated MRSA biofilms under the tested conditions.

## 4. Discussion

MRSA remains one of the most important causes of persistent hospital‐ and community‐acquired infections worldwide. The increasing prevalence of MDR strains, together with the remarkable ability of *S. aureus* to establish biofilms on indwelling medical devices, has substantially reduced the effectiveness of conventional antimicrobial therapies [2, 13, 14]. Biofilm‐associated MRSA infections are particularly difficult to eradicate because bacteria embedded within the EPS matrix exhibit enhanced tolerance to antibiotics and host immune responses [53, 54]. Consequently, there is growing interest in bacteriophages as alternative or adjunctive therapeutics due to their host specificity, self‐replicating nature, and capacity to target biofilm‐associated bacteria [22, 23]. In the present study, we characterized a novel lytic MRSA phage, SA_SGEB_01, isolated from Bangladesh and demonstrated its antibacterial and antibiofilm activity against clinically relevant MRSA strains and catheter‐associated biofilms.

The successful isolation of SA_SGEB_01 using a clinically derived MRSA strain highlights the potential of hospital wastewater as a valuable reservoir of therapeutically relevant bacteriophages. SA_SGEB_01 produced clear plaques and lacked lysogeny‐associated genes, indicating a strictly lytic lifestyle, which is a critical prerequisite for therapeutic phages [55]. Biological characterization further demonstrated efficient replication kinetics, including an optimal MOI of 0.1, a latent period of approximately 45 min, and a burst size of ~91 PFU per infected cell. These characteristics are comparable with those reported for other therapeutically promising members of the genus *Rosenblumvirus*, including phages TSP and vB_SauP_L1 [56, 57], suggesting that SA_SGEB_01 possesses replication properties favorable for rapid bacterial clearance.

Physicochemical stability is another important determinant of phage applicability in therapeutic and biomedical settings. SA_SGEB_01 remained stable across a broad temperature range and retained infectivity from pH 3 to 11. Such environmental robustness is particularly relevant for urinary tract and catheter‐associated applications, where fluctuations in pH and osmolarity are common [20, 58]. The observed stability also supports the potential feasibility of phage storage, handling, and incorporation into therapeutic formulations.

Host range analysis demonstrated that SA_SGEB_01 possesses a moderate but clinically meaningful lytic spectrum. In addition to infecting MRSA, the phage also lysed a methicillin‐resistant *Staphylococcus saprophyticus* isolate, a recognized uropathogen frequently associated with urinary tract infections [59]. This balanced host range may offer therapeutic advantages by maintaining specificity toward pathogenic staphylococci while minimizing disruption of commensal microbiota [22]. Similar host range patterns have been observed among related *Rosenblumvirus* phages, although variations in infectivity against coagulase‐negative staphylococci have been reported [60, 61].

The increasing emergence of antibiotic resistance has stimulated interest in phage–antibiotic combination therapies as a strategy to improve antibacterial efficacy and reduce resistance development [25, 62, 63]. In the present study, SA_SGEB_01 demonstrated synergistic interactions with ciprofloxacin based on FICI analysis despite the ciprofloxacin‐resistant phenotype of the host strain. Although the additional growth inhibition observed in the checkerboard assay was modest at some concentrations, the calculated FICI values met the accepted criterion for synergy. Combined treatment consistently produced substantially greater growth inhibition than either agent alone, with FICI values below 0.5 confirming true synergy. Here, ciprofloxacin was selected because the MRSA isolate exhibited resistance to this antibiotic based on antimicrobial susceptibility testing. Besides, similar synergistic interactions between staphylococcal phages and fluoroquinolones have been reported previously [64, 65], potentially resulting from phage‐mediated disruption of bacterial physiology, increased membrane permeability, or enhanced antibiotic penetration. These findings further support the potential utility of phage–antibiotic combinations against MDR MRSA infections.

A major strength of this study is the demonstration of potent antibiofilm activity by SA_SGEB_01 under both *in vitro* and clinically relevant conditions. In microtiter plate assays, the phage significantly reduced biomass in both immature and mature MRSA biofilms in a dose‐dependent manner. More importantly, SA_SGEB_01 significantly reduced biofilm mass formed on silicone Foley catheter surfaces, producing an approximately 2‐log_10_ reduction in viable bacterial counts. In contrast, vancomycin treatment did not significantly reduce viable cells under the same conditions. These observations are consistent with previous reports showing that conventional antibiotics frequently exhibit limited activity against mature biofilms, whereas bacteriophages can penetrate biofilm matrices and progressively dismantle bacterial communities [30, 66, 67]. Given the increasing burden of catheter‐associated infections, the significant antibiofilm activity of SA_SGEB_01 highlights its potential relevance for device‐associated MRSA control.

Genomic characterization further supported the therapeutic suitability of SA_SGEB_01. The phage possessed a compact double‐stranded DNA genome lacking genes associated with lysogeny, antibiotic resistance, or known bacterial virulence factors, which is an essential safety consideration for clinical phage development [55]. Comparative genomic and phylogenetic analyses consistently placed SA_SGEB_01 within the family Rountreeviridae and the genus *Rosenblumvirus*, with closest relatedness to *Staphylococcus* phage *SCH1*. Pangenome analysis further revealed a conserved genomic backbone together with a diverse accessory gene pool, suggesting ongoing adaptive evolution among related staphylococcal phages [68].

Analysis of lysis‐associated proteins revealed substantial diversity among endolysins but strong conservation among holins within Cluster 5 phages. Most endolysins possessed modular architectures containing CHAP or amidase catalytic domains linked to SH3‐type cell wall–binding domains, which are commonly associated with efficient cleavage of staphylococcal peptidoglycan [68, 69]. In contrast, all holins belonged to the conserved Phage_holin_4_1 superfamily, suggesting stronger evolutionary constraints on membrane permeabilization functions. The greater diversification observed among endolysins may reflect adaptive evolution driven by host cell wall variability and could have implications for the development of engineered lysins or phage‐derived antibiofilm therapeutics.

## 5. Conclusion

This study reports, for the first time, a biologically and genomically characterized *S. aureus* bacteriophage from Bangladesh with demonstrated activity against MDR MRSA biofilms. Phage SA_SGEB_01 exhibited favorable biological characteristics, including efficient lytic activity, broad physicochemical stability, and synergistic antibacterial effects in combination with ciprofloxacin. In addition to its strong activity against planktonic MRSA cells, SA_SGEB_01 significantly reduced both immature and mature biofilms, including catheter‐associated biofilms formed on silicone surfaces. Genomic analysis further confirmed its therapeutic suitability through the absence of lysogeny‐associated, antibiotic resistance, and virulence‐related genes. Collectively, these findings highlight the potential of SA_SGEB_01 as a promising candidate for the control of device‐associated MDR MRSA infections. Future studies should focus on *in vivo* validation, safety assessment, and the development of phage‐based therapeutic formulations for clinical applications.

## Author Contributions

Raphael Kabir Niloy and Shakhinur Islam Mondal contributed equally to this work.

## Funding

This study was funded by the Shahjalal University of Science and Technology, 10.13039/501100007944, LS/2024/2/21, and the University Grants Commission of Bangladesh, 10.13039/100015747, 37.01.0000.073.04.030.23.2135.

## Ethics Statement

The authors have nothing to report.

## Conflicts of Interest

The authors declare no conflicts of interest.

## Supporting Information

Additional supporting information can be found online in the Supporting Information section.

## Supporting information


**Supporting Information 1** Figure S1: Soft agar overlay plate of purified *S. aureus* phage. Plaque size is 2.03 ± 0.2 mm.

**Supporting Information 2** Figure S2: VIRIDIC‐based intergenomic similarity heatmap of SA_SGEB_01 and its nearest *Staphylococcus* phages. Genomic similarity analysis shows the highest similarity with *Rosenblumvirus SCH1* (Accession: KY000084).

**Supporting Information 3** Figure S3: PhamClust pangenome clustering of nonredundant *Staphylococcus* phage genomes. A total of 625 *Staphylococcus* phages were clustered, depending on the PEQ, into 23 clusters, including 17 singletons.

**Supporting Information 4** Table S1: Bacterial strains used in this study.

## Data Availability

The genome sequence data supporting the findings of this study have been deposited in the GenBank Nucleotide Database under accession number PX974350. The associated raw sequencing data are available through the National Center for Biotechnology Information (NCBI) under the following accession numbers: BioProject: PRJNA1392099, BioSample: SAMN54888515, SRA Experiment: SRX31950253, and SRA Run: SRR37000620. Any additional data not included within this article are available from the corresponding authors upon reasonable request.
