# Supplementary material for: Characterization and Therapeutic Potential of a Novel Bacteriophage Targeting Methicillin‐Resistant Staphylococcus aureus Biofilms on Medical Devices
Source: Int J Microbiol. 2026 Aug 28;2026:1281458. doi: 10.1155/ijm/1281458 (PMC13523607; doi:10.1155/ijm/1281458)
Supplement: Supplementary file 1 [file IJM-2026-1281458-s004.docx]

**Supplementary Table 1:** Bacterial strains used in this study.

| **No** | **Species** | **Strain** | **Source** |
| --- | --- | --- | --- |
| 1 | *Staphylococcus aureus* (Isolation Host) | MAD918 | Clinical |
| 2 | *Staphylococcus aureus* | ATCC_25923 | Clinical |
| 3 | *Staphylococcus aureus* | MAD557 | Clinical |
| 4 | *Staphylococcus aureus* | MAD895 | Clinical |
| 5 | *Staphylococcus auricularis* | MAD371 | Clinical |
| 6 | *Staphylococcus epidermidis* | MAD342 | Clinical |
| 7 | *Staphylococcus haemolyticus* | ICD002 | Clinical |
| 8 | *Staphylococcus saprophyticus* | MAD286 | Clinical |
| 9 | *Acinetobacter baumannii* | ATCC_12156 | Clinical |
| 10 | *Acinetobacter baumannii* | ICD005 | Clinical |
| 11 | *Acinetobacter baumannii* | MAD807 | Clinical |
| 12 | *Acinetobacter baumannii* | MAD994 | Clinical |
| 13 | *Acinetobacter lwoffii* | MAD546 | Clinical |
| 14 | *Enterobacter cloacae complex* | ICD003 | Clinical |
| 15 | *Enterococcus faecalis* | EF_BMB_02 | Environmental |
| 16 | *Escherichia coli* | ATCC_25922 | Clinical |
| 17 | *Escherichia coli* | ICD004 | Clinical |
| 18 | *Escherichia coli* | EC_NR_01 | Clinical |
| 19 | *Klebsiella aerogenes* | MAD685 | Clinical |
| 20 | *Klebsiella pneumoniae* | ICD001 | Clinical |
| 21 | *Klebsiella pneumoniae* | MAD058 | Clinical |
| 22 | *Klebsiella pneumoniae* | MAD748 | Clinical |
| 23 | *Klebsiella pneumoniae* | MAD787 | Clinical |
| 24 | *Pseudomonas aerugenosa* | ATCC_27853 | Clinical |
| 25 | *Salmonella enterica* | cc2 | Poultry |
| 26 | *Salmonella enterica* | MD1 | Poultry |
| 27 | *Salmonella enterica* | SAU_S15 | Poultry |
| 28 | *Salmonella enterica serotype Typhi* | ATCC_14028 | Poultry |
| 29 | *Salmonella enterica serotype Typhi* | ST_BMB_01 | Environmental |
| 30 | *Vibrio cholerae* | VC_BMB_01 | Environmental |
